# ASSOCIATION BETWEEN ENVIRONMENTALLY FRIENDLY DIET AND LOWER HEALTH CARE EXPENDITURES

**DOI:** 10.1093/geroni/igz038.2692

**Published:** 2019-11-08

**Authors:** Chin-Lon Lin, Tina H Chiu, Jen-Hung Huang, Ming-Nan Lin

**Affiliations:** 1 Dalin Tzu Chi Hospital, Min-Sheng, Chiayi, Taiwan (Republic of China); 2 Fu Jen Catholic University, Xinzhuang, New Taipei, Taiwan (Republic of China); 3 Hualien Tzu Chi Hospital, Hualien, Hualien, Taiwan (Republic of China)

## Abstract

Vegetarian diets have decreased incidence rates of chronic non-communicable diseases that have been associated with burden on medical systems worldwide. Previous studies propose a relationship between meat consumption and high medical expenditure; however, there is a dearth in the literature with strong evidence supporting the hypothesis between two factors. Using the data from Taiwan’s National Health Insurance Database this study is the first one comparing medical expenditure between vegetarian and non-vegetarian groups among older adults in Taiwan. In the National Health Insurance Database there were 2127 vegetarians matched with 4254 non-vegetarians based on age and gender from 2005 to 2014. Vegetarian diet is associated with approximately 10 – 15% lower total and outpatient medical expenditure. Vegetarians had lower medical expenditure incurred by diabetes and metabolic diseases, mental diseases, and genitourinary diseases. Implication for future research will be discussed at the presentation.

